# Determining the Emotional Intelligence and Compassion Fatigue Among Postgraduate Dental Students Using the Emotional Intelligence Scale and the Quality of Life Scale: An Observational Study

**DOI:** 10.7759/cureus.64060

**Published:** 2024-07-08

**Authors:** Smruti S Saoji, Priyanka Paul, Priyanka D Sontakke, Amit Reche, Srushti Awghad

**Affiliations:** 1 Public Health Dentistry, Sharad Pawar Dental College and Hospital, Datta Meghe Institute of Higher Education and Research, Wardha, IND

**Keywords:** professionals, compassion satisfaction, postgraduate dental students, emotional intelligence, compassion fatigue

## Abstract

Introduction

Compassion fatigue (CF) is a problem for professionals who work with traumatized individuals. CF manifests as reliving personal sorrow, nightmares, flashbacks, intrusive thoughts, numbing or avoiding memories of the experience, and elevated physiological arousal. CF makes professionals more likely to make bad choices, such as misplacing treatments or mistreating patients. Emotional intelligence (EI) is essential to comprehending human behavior, performance, and well-being. EI is a crucial component of professional competency in medical education, as postgraduate dental students are also at risk of having CF. They are professionals and have to work with traumatized patients, so the study was conducted to evaluate their CF and EI. This study aimed to evaluate EI and CF among postgraduate dental students at Sharad Pawar Dental College and Hospital, Wardha, India.

Materials

The observational study was conducted at Sharad Pawar Dental College and Hospital among postgraduate dental students. The data was collected from 80 postgraduate students who responded to the Professional Quality of Life Version 5 and the Emotional Intelligence Scale questionnaires.

Result

A total of 80 postgraduate dental students responded to the Professional Quality of Life Version 5 and the Emotional Intelligence Scale questionnaires, and all the responses were included in the study. Findings revealed that postgraduate dental students treating patients have high levels of CF and EI.

Conclusion

This study indicates that there is a high level of CF and EI in postgraduate dental students. As high CF is harmful and can affect physical and mental well-being, proper management should be done through psycho-education and self-care.

## Introduction

Compassion fatigue (CF) is the term used to describe when someone becomes so tense and concentrated on the suffering of the people they are helping that it becomes traumatizing for them. This specific stress response affects the professional's ability to be empathetic [1]. Johnson states that CF is the outcome of a career in which one becomes emotionally exhausted from one's profession and becomes agitated due to conflict at work [2]. According to Figley (2002), empathy for and exposure to clients who are suffering can lead to CF [3]. CF manifests as reliving personal sorrow, nightmares, flashbacks, intrusive thoughts, numbing or avoiding memories of the experience, and elevated physiological arousal [4]. Burnout relates to the work environment, whereas CF is associated with a professional's involvement with a client's traumatic content [5-7]. It continues to be a widespread occurrence that impacts about half of the individuals who offer assistance to clients in need [8]. CF makes professionals more likely to make bad choices, such as misplacing treatments or mistreating patients [9]. CF in dentists is more likely to occur when they are continuously exposed to traumatic incidents involving their patients [10].

High emotional intelligence (EI) is necessary for professionals who work with vulnerable populations to retain their professional and personal vigor and to provide clients with effective and efficient treatment. The four components of EI are the ability to perceive, comprehend, control, and utilize emotions. These components are accepted by all studies, although some, such as Kabunga et al. (2020), define them differently [11]. EI rose to prominence as a result of Gardner's (1984) conceptions of intrapersonal and interpersonal intelligence, which are, respectively, knowing one's own emotions and understanding others' emotions [12]. Bar-On defines EI as the "array of personal, emotional, and social abilities that enables one to cope with environmental demands" [13]. Within the last 15 years, EI has gained significant attention in scientific circles. The first publications on EI date back to Thorndike's (1920) conceptualization of social intelligence, which is closely related to EI and maybe two highly related parts of the same construct, more commonly known as emotional and social intelligence [13-15]. Emotional regulation necessitates self-awareness, the capacity to access and produce emotions, comprehend and rationalize them, and reflectively control them. It also entails being responsive to societal limitations. EI is sometimes considered a subset of social intelligence, which is defined by some researchers, such as Hedlund and Sternberg (2000), as perceptiveness in interpersonal interactions and the ability to act on that understanding [16]. Different quality-of-life indices and questionnaires are used by authors for research [17,18]. This study aims to evaluate the correlation between EI and CF among dental postgraduate students.

## Materials and methods

The study was approved by the Institutional Ethics Committee, with the research reference number DMIHER (DU)/IEC/2024/79. The study was conducted in Sharad Pawar Dental College and Hospital, Wardha, India, among postgraduate dental students. They were provided with a questionnaire, which experts validated, and a reliability assessment was done using Cronbach's alpha (0.70). A 33-item Emotional Intelligence Scale was used as a tool of assessment [19]. A five-point response format was used. According to the standards, each of the respondents' EI domains was graded as low, moderate, or high. CF was measured by the Compassion Satisfaction and Fatigue Professional Quality of Life Scale (PROQOL) Version 5 [20]. The questions were asked to postgraduate students, and based on their responses on a scale of 1 to 5, further assessment was conducted. Using this scale, compassion satisfaction, burnout, and secondary trauma stress were calculated for each postgraduate dental student.

The questionnaire consisted of demographic details of EI and CF [19,20]. Respondents were contacted by phone and, occasionally, in person. The data was collected through Google Forms. The respondents were informed to fill in the questions according to the given instructions.

Inclusion criteria

Postgraduate dental students were included in this study. Students in the age group of 25 to 35 years were selected for the study. The voluntarily participating postgraduate dental students were included in this study.

Exclusion criteria

Undergraduate dental students were excluded from the study. Postgraduate dental students over 35 years of age were not involved in the study. Uncooperative postgraduate dental students were also excluded from the study.

Sample size calculation

Considering a population size of 100 dental postgraduate students for the survey, with an estimated error of 5% (0.05), the minimum sample size required was calculated using the formula n≥N/1+N(d)^2^. Substituting the values, N=100 and d=0.05, we get 100/1+100(0.05)^2^=80. Therefore, based on this calculation, a minimum sample size of 80 participants was deemed necessary for the study.

Statistic analysis 

Statistical analysis was done by IBM SPSS Statistics for Windows, Version 23 (Released 2015; IBM Corp., Armonk, New York, United States). Descriptive statistics was done to evaluate the mean and standard deviation. Frequency and percentage distribution of the demographic variables were used and chi-square statistics were used to determine the association of EI with CF ( p<0.05).

## Results

A total of 80 postgraduate dental students responded to the questionnaire, and all the responses were included in the study. Table 1 shows the demographic details of participants. The mean age of participants was 25.3±6.12 years. Male participants constituted 26.3%, whereas 73.8% were females.

**Table 1:** Mean and frequency distribution of demographic variables

Demographic variables	Mean	Std. deviation
Age	25.37	6.12
Gender (frequency %)		
Male	21	26.3%
Female	59	73.8%

Table 2 shows the emotional quotient questionnaire, which includes the total mean, mean percentage, and standard deviation for questions 1 to 33.

**Table 2:** Emotional intelligence EQ: Emotional intelligence question

EQ. No	Question	Mean	Mean percentage	Std. deviation
1	When to speak about personal problems	3.72	74.5%	1.05
2	Remember a similar time I faced	4.02	80.5%	0.81
3	I will do well on things I try	3.58	71.75%	1.07
4	Find easy comfort in me	3.70	74%	1.19
5	It is hard to understand the non-verbal message	3.05	61.01%	1.04
6	Re-evaluate what is important	4.17	83.5%	1.02
7	New possibilities	3.45	69%	1.00
8	Emotion makes it worth living	3.63	72.75%	0.97
9	I am aware of my emotions	3.80	76%	0.99
10	Good things to happen	4.05	81%	0.87
11	Like to share emotions	3.33	66.75%	2.53
12	Experience positive emotions	3.58	71.75%	0.92
13	I arrange events	3.42	68.5%	1.01
14	Seek out activities	3.92	78.5%	0.74
15	Aware of non-verbal message	3.46	69.35%	0.98
16	Makes a good impression on others	3.53	70.75%	1.06
17	Solving problems is easy	3.96	79.25%	0.84
18	Recognize people emotions	3.78	75.75%	0.89
19	Emotion changes	3.47	69.5%	0.89
20	Come up with new idea	3.92	78.5%	0.80
21	Have control over emotion	3.35	67%	1.09
22	Easily recognize emotion	3.67	73.5%	0.88
23	Motivate myself	3.93	78.75%	0.66
24	Compliments others	4.17	83.5%	0.72
25	Aware of nonverbal messages others send	3.52	70.5%	0.79
26	Feel as tough as I have experienced it	3.57	71.5%	0.85
27	Feel a change in emotion	3.55	71%	0.85
28	Give up because of failure	2.56	51.25%	1.14
29	Know others feeling	3.64	72.91%	0.83
30	Help people to feel good	4.03	80.75%	0.62
31	Use a good mood to help	3.93	78.73%	0.60
32	Can tell people's feelings	3.81	76.25%	0.74
33	It is difficult to understand people's feelings	3.13	62.75%	0.99

EQ. 1 evaluates an individual's understanding of when it's suitable to talk about personal matters. Respondents generally agree that timing is important when discussing personal difficulties, with a mean score of 3.72 and a standard deviation of 1.05. Although most people believe that timing is important, there are differences in ideas about what constitutes optimal timing, as indicated by the moderate standard deviation. EQ. 2 measures an individual's ability to remember comparable previous events. Respondents strongly agree that they can recall similar situations they had encountered in the past, with a mean score of 4.02. The significant amount of agreement among responders, as suggested by the low standard deviation of 0.81, implies that this ability is widely acknowledged and accepted. Self-efficacy beliefs, or the conviction that one can succeed, are reflected in EQ. 3. Respondents generally demonstrate confidence in their abilities, with a mean score of 3.70. The comparatively high standard deviation of 1.19, however, points to some variation in respondents' confidence levels, with some being more confident than others. EQ. 4 evaluates a person's capacity to comfort themselves. The average score of 3.70 suggests that most respondents find it reasonably simple to console themselves. The high standard deviation of 1.19, however, indicates that opinions differ greatly and that some people find it easier to be comfortable with themselves than others. EQ. 5 assesses how challenging it is to decipher nonverbal cues. Respondents' mean score of 3.05 indicates uncertainty about how hard it is to grasp nonverbal messages. Respondents' levels of difficulty varied, with some finding it more challenging than others, based on the moderate standard deviation of 1.04. EQ. 6 evaluates a person's capacity for reassessing priorities. Respondents strongly agree that people can reassess what is important to them, with a mean score of 4.17.

High agreement among responders, as indicated by the low standard deviation, suggests that this ability is highly appreciated and acknowledged. An individual's receptivity to new opportunities and concepts is shown by this assertion (EQ. 7). Respondents broadly agree that they are receptive to new opportunities, with an average score of 3.45. Certain respondents may have been more open-minded than others, based on the moderate standard deviation of 1.00, indicating some variety in openness across respondents. The importance of emotions in life is examined in EQ. 8. With a mean score of 3.63, most respondents concur that feelings have a role in what makes life worthwhile. The moderate standard deviation indicates that opinions vary, and some people value emotions more than others. The mean value observed for EQ. 24 is the highest, 4.17, while EQ. 5 has the lowest mean value, 3.05. EQ. 31 has the lowest SD (0.60), and EQ. 4 has the highest SD (1.19).

Table 3 describes the mean, mean percentage, and SD of CF. CQ. 12 has the highest mean (4.16), whereas CQ. 28 has the lowest mean (2.80). CQ. 7 has the highest SD (1.21), whereas CQ. 2 has the lowest SD (0.80).

**Table 3:** Compassion fatigue CQ: Compassion fatigue question

CQ. No	Question	Mean	Mean percentage	Std. deviation
1	Happy	3.51	70.2%	0.85
2	Preoccupied with people	3.67	73.4%	0.80
3	Satisfaction to treat	3.83	76.6%	1.04
4	Feel connected	3.27	65.4%	0.85
5	Startled by sounds	3.13	62.6%	1.05
6	Feel invigorated after working	3.22	64.4%	1.13
7	Difficult to separate personal and professional life	2.85	57%	1.21
8	Not productive at work	3.13	62.6%	1.05
9	Affected by traumatic tress	2.92	58.4%	1.14
10	Trapped by job	2.86	57.2%	1.15
11	Felt on edge	3.37	67.4%	0.93
12	Like my work	4.16	83.2%	0.86
13	Feel depressed	3.21	64.2%	1.07
14	Experiencing trauma, I treat	3.10	62%	1.05
15	Beliefs sustain me	3.56	71.2%	0.93
16	Keep up with technique	3.52	70.4%	0.87
17	The person I always wanted to be	3.42	68.4%	0.93
18	Feel satisfied	3.71	74.2%	0.94
19	Feel worn out	3.31	66.2%	1.16
20	Happy thoughts	3.90	78%	0.82
21	Feel overwhelmed	3.57	71.4%	0.86
22	Difference through work	3.69	73.8%	0.83
23	Avoid activities	3.05	61%	1.11
24	Proud of work	3.81	76.2%	0.84
25	Intrusive, frightening thoughts	3.00	60%	1.09
26	Feel bogged down	3.30	66%	1.02
27	Thoughts of success	3.37	67.4%	0.93
28	Can't recall work	2.80	56%	1.09
29	Caring person	3.79	75.8%	0.82
30	Happy to do the work	4.03	80.6%	0.81

Highest CF

CQ. 10 (trapped by job) has the highest mean score at 2.86, suggesting that the respondents felt trapped by their job. An elevated score indicates that people might have limitations or constraints in their work duties, which could result in burnout or exhaustion. CQ. 7 (hard to keep personal and work lives apart), with a mean score of 2.85, indicates that respondents find it difficult to keep their personal and work lives apart. People who find it difficult to draw boundaries between their personal and professional lives may experience higher levels of stress, which could promote CF. CQ. 9 (traumatic stress affected), with a mean score of 2.92, suggests that respondents are impacted by traumatic stress to some extent. Traumatic stress can have a detrimental effect on one's emotional health and exacerbate CF, particularly for individuals who work in professions where trauma is a common occurrence.

Lowest CF

CQ. 12 (like my work): Respondents frequently indicate a strong liking for their work, with a high mean score of 4.16. This suggests that the person is highly fulfilled and satisfied in their work, which may serve as a buffer against CF. People who find fulfillment in their profession may be more resilient and driven when faced with difficult situations. CQ. 30 (happy to do the work): The high mean score of 4.03 for this question also indicates that respondents are content with their work. Like CQ. 12, high levels of job satisfaction and contentment lead to lower levels of CF because they improve general well-being and create a positive work environment. CQ. 24 (proud of work): Respondents feel generally proud of their work, with a mean score of 3.81. Reliability and resilience in the face of difficult circumstances are fostered when one feels proud of and accomplished in one's professional endeavors, which can act as a preventive measure against CF. The areas where respondents may be more susceptible to or resistant to CF are highlighted by these highest and lowest CF ratings, which together offer insights into potential areas for support or intervention.

Table 4 shows the mean and mean percentage distribution of CF and EI. The total mean distribution of EI is 119.98±12.54, and CF is 101.88±14.00. The mean percentage of EI is 72.71%, and CF is 67.92%. There is a positive correlation between EI and CF, r=0.087 (p=0.44), with a statistically significant difference observed in their correlation with each other.

**Table 4:** Mean percentage and mean distribution of compassion fatigue and emotional intelligence

Variables	Mean	Mean percentage	Std. deviation	Correlation (r)
Emotional intelligence	119.98	72.71%	12.54	p-value=0.441 (0.087)
Compassion fatigue	101.88	67.92%	14.00	

## Discussion

The purpose of the study was to determine whether CF and EI are related among postgraduate dental students [1]. Two scales were used: the 33-item Emotional Intelligence Scale and the Professional Quality of Life Scale Version 5, to evaluate EI and CF in this study.

CF, described as the formal caregiver's diminished ability or desire to empathize with others or "carry the suffering of clients," aligns with current usage [21,1,3]. CF is also defined as "the natural consequence behavior and feelings arising from knowing about a traumatic incident that a person goes through or experiences" [21]. CF manifests as reliving personal sorrow, nightmares, flashbacks, intrusive thoughts, numbing or avoiding memories of the experience, and elevated physiological arousal [4]. In the early 1990s, Salovey and Mayer introduced the concept of EI, defining it as "an aspect of social intelligence that entails having the capacity to recognize, identify, and monitor oneself and others' feelings to inform decisions and behavior" [22]. EI is crucial for understanding human behavior, performance, and well-being [23,24], and has been highlighted as essential for professional competency in medical education [25,26]. According to Wagner et al. (2002), doctors' EI is linked to better patient satisfaction [27].

Kabunga et al. (2020) conducted a similar study on EI and CF among psychotherapists. They observed an inverse relationship between the symptoms of CF and four components of EI: social consciousness, self-management, awareness of oneself, and social competencies. These correlations were statistically significant at p<0.0001. The development of psychotherapists' EI can help them better control their emotions, thereby preventing CF and protecting their physical and mental health [11]. Jacobson (2012) conducted a similar study on the risk of CF and burnout, as well as the potential for compassion satisfaction among employee assistance professionals. The study found that the model was significant (F=3.83, p<0.001), with an overall variance of 21.5% in predicting the likelihood of CF. Theoretically, while CF and satisfaction are common avoidable responses to working with traumatized people, burnout is a more severe, long-lasting reaction [28].

Choi (2011) conducted a study on the organizational impacts on the secondary traumatic stress of social workers. They observed that social workers with higher levels of support from their teams, bosses, and peers experienced a reduction in secondary traumatic stress and CF [29]. Salovey and Mayer (1990) conducted a contradictory study on EI. The observation in this study was that emotionally intelligent individuals are capable of identifying and expressing their own feelings, controlling affect, identifying the emotions of others, and using emotions to drive adaptive behavior [22].

Table 5 indicates the different studies done by authors using various methods on CF and EI and have achieved different results in their studies.

**Table 5:** Different studies done by authors on CF and EI CF: Compassion fatigue; EI: Emotional intelligence; STS: Secondary traumatic stress; OLS: Ordinary least squares

Sr. No	Author	Methods	Results
1	Kabunga et al. (2020) [11]	The study design used was cross-sectional correlational, and quantitative methodologies were used.	Psychotherapists need to become more emotionally intelligent in order to manage their emotions better, which will directly reduce compassion fatigue and safeguard their mental and physical well-being.
2	Jacobson (2012) [28]	This study evaluated the possibility of compassion fatigue using a cross-sectional, one-group survey approach.	According to the study, dealing with individuals who have experienced trauma can naturally lead to compassion fatigue and satisfaction, and these symptoms can be avoided or reversed.
3	Choi (2011) [29]	The study employed a multivariate OLS linear regression model to regress STS on three variables: job circumstances, organizational support, and demographic/control characteristics.	Those who enjoy strong social support showed reduced degrees of compassion fatigue.
4	Salovey and Mayer (1990) [22]	The appraisal and expression of emotion have been overlooked when measuring mental abilities.	Considerations relating emotional intelligence to the individual are studied.

The result of this study shows both a high level of CF and a high level of EI. This indicates that it is possible for some postgraduate dental students to feel a high level of CF despite having a high level of EI. As a high level of CF has been observed in the study, it is imperative to manage their psychological well-being and to better treat patients. A person with high CF needs compassion satisfaction and self-care [30]. It is thought that by creating healthy coping strategies and resources to handle upsetting and stressful situations encountered at work, compassion satisfaction helps professionals advance professionally and enhance their physical, mental, and emotional well-being [31].

Limitations

This study was limited to dental students. Because of the smaller sample size, it was not possible to determine a causal relationship between CF and EI. The study was completely dependent on the respondent's results.

## Conclusions

This study indicates that postgraduate dental students treating patients can have a high level of CF and EI. High CF is harmful and can affect their physical and mental well-being, which is detrimental to both the students and the patients. Therefore, proper management should be implemented through psychoeducation and self-care. Additionally, appropriate job design and management should be undertaken for the betterment of mental health.

## Figures and Tables

**Figure 1:**
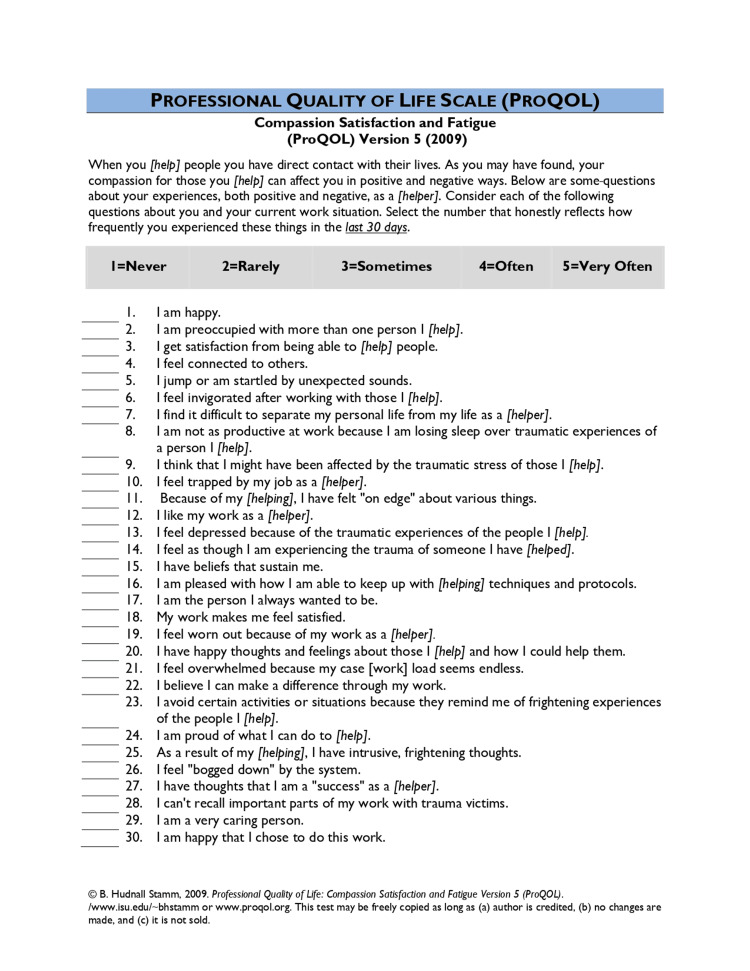
Professional Quality of Life Scale (PROQOL) questionnaire Image source: [20]

**Figure 2:**
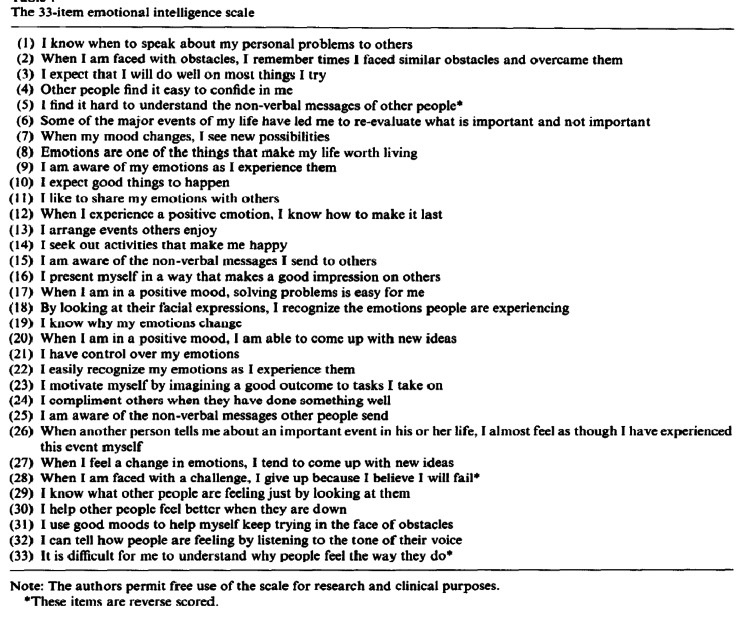
Emotional Intelligence Scale questionnaire Image source: [19]

## References

[1] Figley CR (2002). Compassion fatigue: psychotherapists' chronic lack of self care. J Clin Psychol.

[2] Borhani F, Abbaszadeh A, Nakhaee N, Roshanzadeh M (2014). The relationship between moral distress, professional stress, and intent to stay in the nursing profession. J Med Ethics Hist Med.

[3] Figley CR (2002). Treating Compassion Fatigue. https://books.google.co.in/books?hl=en&lr=&id=2qyVRQ8y7SkC&oi=fnd&pg=PP1&dq=Introduction.+In+C.+Figley+(Ed.),+Treating+compassion+fatigue+(pp.+1-14).+New+York:+Brunner-Routledge&ots=XNFFhLr1MI&sig=b_jZA9O_PIX1fZrcdYlRA3x3Q50&redir_esc=y#v=onepage&q&f=false.

[4] Stamm B (2010). The Concise ProQOL Manual: The Concise Manual for the Professional Quality of Life Scale. https://d1wqtxts1xzle7.cloudfront.net/62440629/ProQOL_Concise_2ndEd_12-201020200322-88687-17klwvb-libre.pdf?1584952980=&response-content-disposition=inline%3B+filename%3DThe_Concise_ProQOL_Manual.pdf&Expires=1717218212&Signature=UP5suImTr46RsFCuksdKuxy3yK0WloIcfdDZi8Tfr5h9wPDUBtYdKdK6crPQyfnImnHNZ7l6~nVTHLfe2CCIovoSOYKL--fZg6MmMJVwHVT7eLIwznkmMPliXfvrlAvxcfbRPcdwwt5SjgIskyIs5ZD8Vm7wJPaZ0Y76Et-ZUW2pB0cMTfJjg85p77ERPmaiMA8sD1c3-Of7evOxbAxS5jIL-Ycgtuyh4Q5ZMCK9F00atEmKQcu4QeQ2FgoYcaY1KczffbHbvUg19ttW-JGZARmqWp3UIxu75iPQ5sJv3S9FnVP~jAaqz8IxGnFxcQ~IOcCKiDIezvsjb5L2wQazIQ__&Key-Pair-Id=APKAJLOHF5GGSLRBV4ZA.

[5] Collins S, Long A (2003). Working with the psychological effects of trauma: consequences for mental health-care workers--a literature review. J Psychiatr Ment Health Nurs.

[6] Maslach C (2003). Burnout: The Cost of Caring. Ishk.

[7] Pryce JG, Shackelford KK, Pryce DH (2007). Secondary Traumatic Stress and the Child Welfare Professional. https://doi.org/10.1093/oso/9780190615918.001.0001.

[8] Injeyan MC, Shuman C, Shugar A, Chitayat D, Atenafu EG, Kaiser A (2011). Personality traits associated with genetic counselor compassion fatigue: the roles of dispositional optimism and locus of control. J Genet Couns.

[9] Drury V, Craigie M, Francis K, Aoun S, Hegney DG (2014). Compassion satisfaction, compassion fatigue, anxiety, depression and stress in registered nurses in Australia: phase 2 results. J Nurs Manag.

[10] Hinderer KA, VonRueden KT, Friedmann E, McQuillan KA, Gilmore R, Kramer B, Murray M (2014). Burnout, compassion fatigue, compassion satisfaction, and secondary traumatic stress in trauma nurses. J Trauma Nurs.

[11] Kabunga A, Anyolitho MK, Betty A (2020). Emotional intelligence and compassion fatigue among psychotherapists in selected districts of Northern Uganda. S Afr J Psychol.

[12] Walkley AB (1984). Frames of Mind: The Theory of Multiple Intelligences. Basic books.

[13] Ashforth BE (2000). The handbook of emotional intelligence: theory, development, assessment, and application at home, school, and in the workplace. Pers Psychol.

[14] Bar-On R (2003). The Bar-On Emotional Quotient Inventory. https://www.eitrainingcompany.com/wp-content/uploads/2009/04/eqi-133-resource.pdf.

[15] Bar-On R (2004). The Bar-On Model of Emotional-Social Intelligence. Nova Science.

[16] Hedlund J, Sternberg RJ (2000). Too many intelligences? Integrating social, emotional, and practical intelligence. Creat Educ.

[17] Khubchandani S, Bhoyar A, Dahane TM, Sathe S, Godbole S, Pisulkar SK (2021). Masticatory ability and oral health-related quality of life in partially edentulous patients involving posterior teeth after rehabilitation with tooth-supported fixed dental prosthesis. J Pharm Res Int.

[18] Deolia S, Khubchandani S, Gopalan A, Sehgal M, Saoji M, Sen S (2019). Evaluation of oral health-related quality of life in patients undergoing hemodialysis. JORDMIMS.

[19] Schutte NS, Malouff JM, Hall LE, Haggerty DJ, Cooper JT, Golden CJ, Dornheim L (1998). Development and validation of a measure of emotional intelligence. Pers Individ Dif.

[20] (2024). Professional quality of life scale (PROQOL). https://socialwork.buffalo.edu/content/dam/socialwork/home/self-care-kit/compassion-satisfaction-and-fatigue-stamm-2009.pdf.

[21] Rushforth A, Durk M, Rothwell-Blake GA, Kirkman A, Ng F, Kotera Y (2023). Self-compassion interventions to target secondary traumatic stress in healthcare workers: a systematic review. Int J Environ Res Public Health.

[22] Salovey P, Mayer JD (1990). Emotional intelligence. Imagin Cogn Pers.

[23] Muchinsky PM (2000). Emotions in the workplace: the neglect of organizational behavior. J Organ Behav.

[24] Barsade SG, Gibson DE (2007). Why does affect matter in organizations?. Acad Manage Perspect.

[25] Carrothers RM, Gregory SW, Gallagher TJ (2000). Measuring emotional intelligence of medical school applicants. Acad Med.

[26] Pau AK, Croucher R (2003). Emotional intelligence and perceived stress in dental undergraduates. J Dent Educ.

[27] Wagner PJ, Moseley GC, Grant MM, Gore JR, Owens C (2002). Physicians' emotional intelligence and patient satisfaction. Clin Res.

[28] Jacobson JM (2012). Risk of compassion fatigue and burnout and potential for compassion satisfaction among employee assistance professionals: protecting the workforce. Traumatology.

[29] Choi GY (2011). Organizational impacts on the secondary traumatic stress of social workers assisting family violence or sexual assault survivors. Health Soc Care Community.

[30] Harr CR, Brice TS, Riley K, Moore B (2014). The impact of compassion fatigue and compassion satisfaction on social work students. J Soc Soc Work Res.

[31] Vu F, Bodenmann P (2017). Preventing, managing and treating compassion fatigue. Swiss Arch Neurol Psychiatry Psychother.

